# Gestational Diabetes Mellitus per Different Diagnostic Criteria, Risk Factors, Obstetric Outcomes and Postpartum Glycemia: A Prospective Study in Ghana

**DOI:** 10.3390/clinpract11020039

**Published:** 2021-05-07

**Authors:** Faith Agbozo, Abdulai Abubakari, Francis Zotor, Albrecht Jahn

**Affiliations:** 1 Department of Family and Community Health, School of Public Health, University of Health and Allied Health Sciences, Ho, Ghana, Private Mail Bag 31 Ho, Ghana; fbzotor@uhas.edu.gh; 2 Heidelberg Institute of Global Health, University Hospital Heidelberg Germany, Im Neuenheimer Feld 130.3, 60120 Heidelberg, Germany; albrecht.jahn@uni-heidelberg.de; 3 Public Health Department, University for Development Studies, Tamale Ghana, Tamale P.O. Box TL 1350, Ghana; abubakari.abdulai1@uds.edu.gh

**Keywords:** gestational diabetes mellitus, hyperglycemia in pregnancy, blood glucose, pregnancy, prevalence, risk factors, newborn, pregnancy outcome, postpartum period, Ghana

## Abstract

The surge in gestational diabetes mellitus (GDM) globally requires a health system tailored approach towards prevention, detection and management. We estimated the prevalence of GDM using diverse recommended tests and diagnostic thresholds, and also assessed the risk factors and obstetric outcomes, including postpartum glycemia. Using a prospective cohort design, 446 singleton pregnant women without pre-existing diabetes did GDM tests in five hospitals in Ghana from 20–34 weeks using fasting plasma glucose (FPG), one-hour and 2-h oral glucose tolerance test (OGTT). Birth outcomes of 403 were assessed. GDM was diagnosed using six international diagnostic criteria. At 12 weeks postpartum, impaired fasting glucose (6.1–6.9 mmol/L) and diabetes (FPG ≥7.0 mmol/L) were measured for 100 women. Per FPG and 2-h OGTT cut-offs, GDM prevalence ranged between 8.3–23.8% and 4.4–14.3%, respectively. Risk factors included overweight (OR = 2.13, 95% CI: 1.13–4.03), previous miscarriage (OR = 4.01, 95% CI: 1.09–14.76) and high caloric intake (OR = 2.91, 95% CI: 1.05–8.07). Perineal tear (RR = 2.91, 95% CI: 1.08–5.57) and birth asphyxia (RR = 3.24, 95% CI: 1.01–10.45) were the associated perinatal outcomes. At 12 weeks postpartum, 15% had impaired fasting glucose, and 5% had diabetes. Tackling modifiable risk factors is crucial for prevention. Glycemic monitoring needs to be integral in postpartum and well-child reviews.

## 1. Introduction

Research on gestational diabetes mellitus (GDM) dates back to 1882 [1]. However, the Hyperglycemia and Adverse Pregnancy Outcomes (HAPO) study conducted at 15 centers in nine countries to assess the association between varying degrees of maternal glucose and adverse outcomes [2] sparked interest in GDM research and its clinical practice. It formed the foundation for the diagnostic criteria currently recommended by the International Association of Diabetes and Pregnancy Study Group (IADPSG) [3]. GDM accounts for 86.4% of all cases of hyperglycemia in pregnancy [4]. According to the HAPO study, 18–26% of pregnancies are affected by GDM [2], but globally, prevalence is estimated to be between 1–14% [5]. In sub-Saharan Africa, there has been an upward trajectory in prevalence between 2015 [6] and 2019 (8.5%) [7].

Attributed to the rising prevalence are modifiable and non-modifiable risk factors [2,7,8] driven primarily by the demographic, epidemiological, nutrition, obstetric and technological transitions. GDM is linked to adverse maternal and fetal outcomes [2,9,10] as well as long-term cardiometabolic complications [8,11,12]. It is argued that the use of different screening algorithms and lower diagnostic criteria may increase the rates, thereby masking the true prevalence [13]; generating concerns of overdiagnosis [14,15,16] and unnecessary medicalization of pregnancy [17,18]. Overdiagnosis could complicate treatment outcomes [13], cause emotional, physical and financial distress to women diagnosed, increase care providers’ workload as well as healthcare expenditure [19,20].

**Figure 1:**
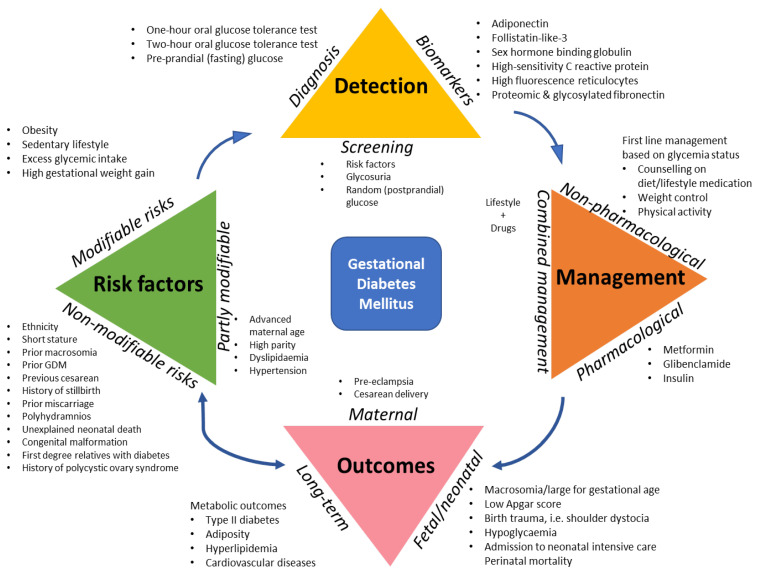
Conceptual framework showing the current areas of gestational diabetes research. Note: Author designed.

Despite the widened interest in GDM research, studies from low-income settings are sparse and generally narrowed to prevalence and risk factors (Figure 1). Adverse pregnancy outcomes are not well established in these settings, and often, retrospective data from tertiary hospitals are used which do not reflect population-based prevalence. Additionally, there is little focus on diet and pharmacological treatment, obstetric outcomes and future health impacts. Meanwhile, because these health systems are traditionally designed to cater for infectious diseases, their readiness towards managing the surge is weak. Further, many studies fail to indicate the diagnostic approach used (be it universal ‘one-step’ versus selective ‘two-step screening), and the resulting prevalence according to the ‘gold standard’ 2-h oral glucose tolerance test (OGTT) and fasting plasma glucose (FPG) tests.

In Ghana, GDM prevalence per 2-h OGTT ≥8.5 mmol/L (153 mg/dL) is 9.3% [21]. Given that this study was conducted in the largest referral hospital, where 92.5% of the study participants are urban dwellers, findings might not reflect the general population where lower prevalence is expected. Estimating the prevalence per different diagnostic tests and cut-offs and identifying the obstetric outcomes could enhance screening, diagnosis and management modalities. This is especially important at primary and secondary levels of antenatal care (ANC) where specialist care and essential medical supplies are often lacking. Therefore, this study aimed to (1) estimate the prevalence of GDM in lower-level facilities using some common diagnostic cut-offs; (2) assess the risk factors; (3) influence of GDM on perinatal outcomes and (4) maternal glycemic status at 12 weeks postpartum.

## 2. Material and Methods

### 2.1. Study Context and Design

This observational study was conducted as a prospective longitudinal study and reported in line with the STROBE (strengthening the reporting of observational studies in epidemiology) statement for cohort studies.

**Figure 2:**
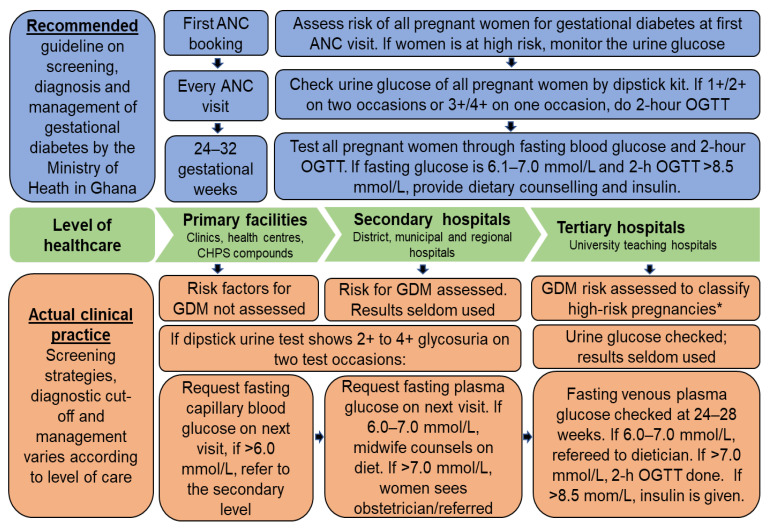
Recommended standard of care for GDM detection in Ghana vis-à-vis the actual clinical practice. Note: Author designed. ANC, antenatal care clinic; 2-h OGTT, two-hour oral glucose tolerance test; GDM, gestational diabetes mellitus; CHPS, Community Health-Based Planning Services.

In Ghana, universal testing of all pregnant women using the ‘one-step’ screening approach is the current guideline for GDM detection [22]. The blue shaded area in Figure 2 illustrates the recommended screening and testing modalities. Essentially, at every ANC visit, urine glucose of all pregnant women is checked. If the urine glucose is 1+/2+ on two occasions or 3+/4+ on any single visit, 2-h OGTT is performed. Between 24–32 gestational weeks, all pregnant women should perform both fasting blood glucose and 2-h OGTT. When the fasting blood glucose is 6.1–7.0 mmol/L or the 2-h OGTT >8.5 mmol/L, GDM is diagnosed. Diet and exercise therapy, which is the first-line management strategy, is initiated, but where glycemic control is unsatisfactory, insulin is administered.

Although the use of oral anti-diabetic medications is contraindicated during pregnancy in Ghana [22], some clinicians administer metformin as a monotherapy or in combination with insulin based on evidence that metformin significantly lowers post-prandial blood glucose than insulin [23]. However, the guideline is silent on the exact glycemic values at which administration of hypoglycemic agent is utterly necessary in situations where diet therapy does not lead to satisfactory glycemic control. Regarding actual clinical implementation, there exist discrepancies at the various levels of healthcare. The screening and management practice in primary, secondary, and tertiary levels of care is shown in the orange shaded area in Figure 2. Despite the national target of 85% pregnant women receiving at least four ANC visits, in 2016, only 72% achieved this target in the study region. ANC booking in the first trimester and skilled delivery were approximately 45% [24]. The study sites have been described elsewhere [25].

### 2.2. Participants

**Figure 3:**
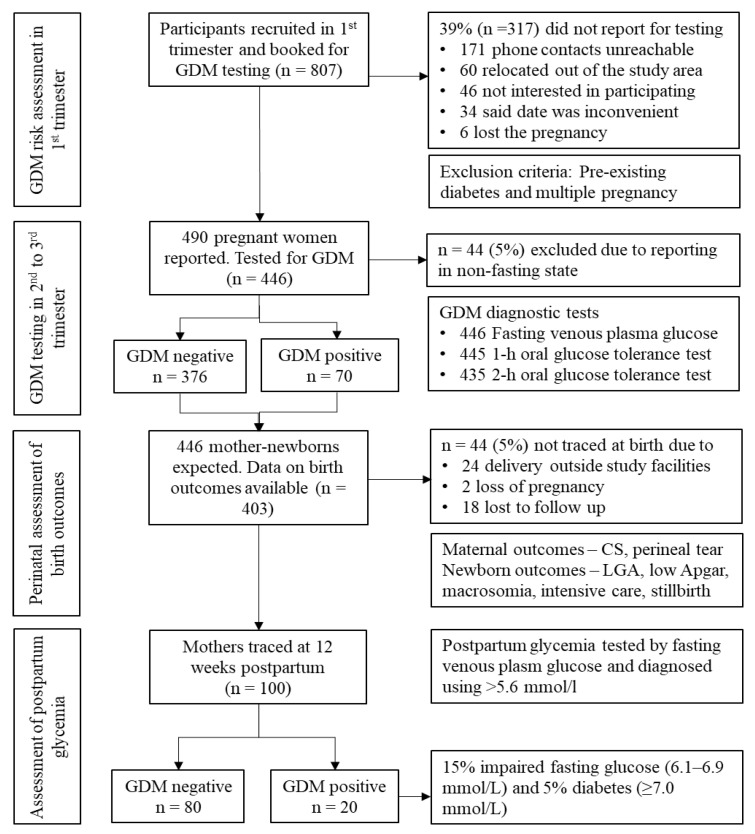
Number of participants followed-up at each stage of the study. Note: *n*, number of participants; GDM, gestational diabetes mellitus; CS, cesarean section; LGA, large-for-gestational-age.

Participants were recruited in the first trimester of pregnancy and the cohort followed-up until 12 weeks postpartum. In line with ANC delivery in Ghana, participants were proportionately allocated to one clinic, three municipal hospitals and one teaching hospital representing primary, secondary and tertiary levels of care respectively, which serve rural and urban communities in the Volta Region, Ghana. The sample size of 416 was determined using GDM prevalence of 9.3% [21], a population of 516,461 women in their reproductive age in the region, a 95% confidence level corresponding to 1.96 Z-score, a 5% error margin and a design effect of 3.2 accounting for variability in the different levels of ANC [25]. Based on 43.7% access to a skilled attendant at birth in Ghana [24], the sample size was increased to 800 to account for any attrition. Singleton pregnant women without pre-existing diabetes who registered for ANC in the first trimester of pregnancy were eligible. At ANC booking, random blood glucose and glycated hemoglobin (HbA1c) were checked. Participants whose random blood glucose values (≥11.1 mmol/L) and HbA1c (≥6.5%, 7.8 mmol/L) were suggestive of pre-existing diabetes were excluded (*n* = 10). Women who did not intend to deliver in any of the study facilities were also excluded. All women (*n* = 3093) who registered for ANC in the first trimester in the five study facilities from June 2016 to April 2017 constituted the sampling frame. Eligible participants were consecutively selected until the required sample size was obtained. Overall, 807 participants were booked for GDM testing, of which 490 reported but about 5% (*n* = 44) arrived in a non-fasting state and were thus excluded. Reasons for dropout from the study are shown in Figure 3. Overall, 446 performed the diagnostic tests, 403 were traced at delivery and 100 were followed-up at 12 weeks postpartum.

### 2.3. Data Collection

#### 2.3.1. Anthropometric, Health and Dietary Indicators

At the first ANC booking, which was typically before the 16th week of gestation, we conducted one-on-one interviews to obtain data on socio-demographic variables. We measured body weight, height and mid-upper arm circumference (MUAC) following standard procedures and derived the body mass index (BMI) from the anthropometric indices. We extracted information from the maternal health record booklet on participants’ obstetric (gravida, parity, previous macrosomic births, cesarean section [CS], miscarriages, perinatal and neonatal deaths) and medical histories (first-degree relations with diabetes and/or hypertension).

We assessed habitual dietary patterns using a food frequency questionnaire (FFQ). The FFQ had a frequency of consumption categories ranging from daily, weekly, fortnightly, monthly, rarely to never. Designed a priori based on frequently consumed foods in Ghana, the FFQ provided qualitative data on food intake, including snacks and beverages. To minimize recall biases, we checked the plausibility of the reported dietary intakes by collecting a non-quantitative 24-h recall data. Daily consumption of any carbohydrate-dense foods that contributed over 70% of the glycemic index (GI) value was assigned a score of one. Based on the cumulative scores, daily intake of five or more foods that contributed over 70% GI value was rated as high caloric intake; daily intake of three to four high GI value foods was considered to be moderate caloric intake, and daily consumption of two or less high GI value foods was considered to be low caloric intake.

During the monthly ANC visits, we took blood pressure, gestational weight gain and urine glucose/protein measurements. MUAC was measured once in each trimester and the cut-off determined using the population median value. Per recommendations from the Institute of Medicine on ideal pregnancy weight gain, a woman was considered to be at high risk for GDM if her body weight for gestational age was above the threshold for her BMI group. The BMI groups and the corresponding pregnancy weight gain categories are underweight (<18.5 kg/m^2^), 12.5–18.0 kg; normal weight (18.5–24.9 kg/m^2^), 11.5–16.0 kg; overweight (25.0–29.9 kg/m^2^), 7.0–11.5 kg; and obese (≥30 kg/m^2^), 5.0–9.0 kg.

#### 2.3.2. GDM Testing and Diagnosis

Testing was scheduled between 20–28 gestational weeks using the one-step universal screening approach. Participants who were unable to report at the designated period were rescheduled between 30–34 weeks (*n* = 178, 36.3%). After 12 h overnight fast, pre-prandial venous blood was drawn from the antecubital fossa to measure the fasting venous plasma glucose (FPG) and lipid profile (total cholesterol, triglycerides; high, low and very-low-density lipoproteins). Thereafter, participants were given 75-g anhydrous glucose dissolved in 300 mL of water at ambient temperature to drink under direct observation. One milliliter blood was collected at one and two hours postprandial following standard operating procedures. Laboratory analysis was done on the fully automated Selectra ProM (Elitech Group, Puteaux, France) clinical chemistry analyzer operating on the kinetic enzymatic peroxidase-antiperoxidase principle. We estimated the prevalence of GDM by applying some major thresholds for GDM diagnosis based on fasting plasma glucose, 1-h and 2-h OGTT values. We followed the criteria of the IADPSG [3] officially adopted by the World Health Organization [19], the International Federation of Gynecology and Obstetrics [8] and the American Diabetes Association [5], as well as cut-offs by the National Institute for Health and Care Excellence [26], the Canadian Diabetes Association [27], the American Congress of Obstetricians and Gynecologists [28] and the protocol employed in Ghana [22]. Since one abnormal value is sufficient to make a diagnosis [3,19], participants without the full GDM test results were included in the analysis and reporting. As this study design was observational, all GDM cases received the usual routine care.

#### 2.3.3. Pregnancy Outcomes

The primary outcome was GDM. At peripartum, obstetric outcomes assessed included CS, perineal tear, postpartum hemorrhage (defined as estimated blood loss above 500 mL), newborn adiposity and survival. We estimated adiposity using three indicators: (1) macrosomia defined as birth weight ≥4 kg regardless of gestational age at birth; (2) large-for-gestational-age (LGA) defined as birth weight >90th percentile per the InterGrowth study standards accounting for gestational age at birth and sex of the newborn; and (3) Ponderal Index (PI) calculated as the birth weight (g)/length (cm^3^) × 100. PI was classified as small-for-gestational-age (<2.0), marginal (2.0–2.5), normal (2.5–3.0.) and large-for-gestational-age (≥3.0). Survival of the newborn was assessed using four indicators: (1) Apgar score at one and five minutes; (2) resuscitation, (3) admission to neonatal intensive care unit (NICU) and (4) perinatal death. Secondary outcomes were gestational age at birth and random glucose of the newborn determined from the capillary blood collected at the heel between one to two hours after birth. At 12 weeks postpartum, we measured FPG of the GDM cases to diagnose impaired fasting glucose (6.1–6.9 mmol/L), and diabetes (FPG ≥7.0 mmol/L) using the International Federation of Gynecology and Obstetrics’ diagnostic criteria for non-pregnant women [8,29].

### 2.4. Statistical Analysis

Descriptive analysis was conducted using unpaired *t*-test and Chi-square test. Differences between the GDM present or absent groups was tested using a dichotomous outcome tabulated in a two-by-two table with the dichotomous input variables. Inferential analysis was conducted using unconditional logistic regression to generate crude estimates of association. Variables that had theoretical evidence of association with GDM or recorded *p* < 0.10 in the crude estimates were included in the adjusted model. To control for confounding variables, multivariate binary logistic regression was modeled and the adjusted odds ratios (aOR) obtained through the Cochran -Mantel-Haenszel statistic. We conducted a simple linear regression to estimate the coefficient of a unit rise in blood glucose on individual pregnancy outcomes assessed. A correlation matrix was computed to identify collinearity and possible confounders, in addition to interaction terms considered in the final model selection. Adjusting for confounding variables in a multivariate analysis, binary logistic regression model was run to estimate the relative risk for an adverse obstetric outcome. Missing values were deleted pairwise. As multiple birth outcomes were tested simultaneously, the effect of multiple comparisons was adjusted for using the Bonferroni correction. A corrected *p* < 0.05 (two-sided) and confidence intervals (CI) excluding one were considered to be associated with the outcome measures. Analysis was done in Stata software (version 14.2).

### 2.5. Ethical Considerations

The Ghana Health Service Ethics Review Committee (GHS-ERC-GM 04/02/16) and the Institutional Review Board of Heidelberg University Medical Faculty (S-042/2016) approved the study. We obtained written informed consent from all study participants, including participants below 18 years who were ethically regarded as emancipated adults.

## 3. Results

### 3.1. Prevalence of GDM

Out of 490 participants who reported for GDM testing, 16.5%, 70.6% and 12.9% used primary, secondary and tertiary facilities, respectively. A third were residing in rural areas. Half were aged 20–29 years; 29.8% were primiparous women whereas 12.8% had more than five pregnancies in their lifetime. Additionally, 63.8% had only primary education while an equal proportion (64.9%) were informal sector workers. In terms of pregnancy intention, 37.1% of the participants did not plan their current pregnancy.

Overall, 446 participants performed fasting plasma glucose test while 445 and 435 completed 1-h and 2-h OGTT, respectively. Seventy of the study participants had at least one pre- or postprandial hyperglycemic glucose value. At the point of delivery and at 12 weeks postpartum, 63 and 20 of those diagnosed were traced, respectively. Among the GDM and non-GDM groups, significant differences were observed in the mean FPG (6.04 vs. 4.28 mmol/L), 1-h OGTT (8.81 vs. 6.41 mmol/L) and 2-h OGTT (8.64 vs. 5.91 mmol/L). Table 1 shows the prevalence of GDM according to some commonly used diagnostic criteria. Per the IADPSG/WHO criteria, participants with 1-h OGTT above 10.0 mmol/L was 4.5%. If the two-step diagnostic criteria of the Canadian Diabetes Association (≥10.0 mmol/L) was applied for 1-h OGTT [27], 3.6% positive cases would be obtained. Two percent (*n* = 10, 95% CI 1.0 to 3.5) of the hyperglycemic cases were overt diabetes first detected in pregnancy.

### 3.2. Risk Factors for GDM

Mean age of the GDM group (29.82 ± 6.80 years) was significantly higher than the non-GDM group (28.19 ± 5.99 years) so was first-trimester body weight (66.38 ± 14.32 vs. 62.37 ± 12.75 kg), BMI (24.77 ± 4.82 vs. 23.21 ± 4.30 kg/m^2^) and MUAC (29.51 ± 4.40 vs. 28.08 ± 3.48 cm) but gestational weight gain (11.67 ± 5.21 vs. 11.06 ± 5.18 kg), blood pressure and lipid profile were statistically similar. Presented in Table 2 is a comparison of the two groups. From the univariable binary regression, we identified maternal age above 35 years, partner’s education up to primary level, ANC in a primary facility, overweight/obese, MUAC >30 cm, history of spontaneous abortion, preeclampsia and habitual intake of high GI foods as independent risk factors for GDM. Adjusting for covariates, overweight/obesity (aOR = 2.13, 95% CI: 1.13–4.03), MUAC >30 cm (aOR = 2.97, 95% CI: 1.31–5.58), history of abortion (aOR = 4.01, 95% CI: 1.09–14.76) and habitual intake of high GI foods (aOR = 2.91, 95% CI: 1.05–8.07) remained associated with the outcome (Table 3).

### 3.3. Perinatal Outcomes

Comparing the GDM (*n* = 63) and non-GDM (*n* = 340) groups traced at delivery, other than CS (31.4% vs. 19.5%) which was significantly higher in the GDM group, proportions of obstructed labor (15.6% vs. 11.1%), episiotomies (11.2% vs. 13.8%), perineal tears (19.4% vs. 11.0%) and macrosomic births (birth weight >4 kg) (4.3% vs. 3.1%) were similar. Comparing the obstetric outcomes in the GDM positive and negative groups, we observed significant differences in the amount of blood lost (228.42 ± 123.48 vs. 178.54 ± 105.89 mL, *p* = 0.010) and birth weight (3.23 ± 0.49 vs. 3.06 ± 0.45 kg, *p* = 0.035). However, birth length (49.59 ± 2.74 vs. 48.87 ± 3.34 cm), head circumference (34.38 ± 1.71 vs. 33.97 ± 2.04 cm), Ponderal Index (2.70 ± 0.49 vs. 2.69 ± 0.61 g/cm^3^) and the newborn’s blood glucose (4.74 ± 0.90 vs. 3.924 ± 1.31 mmol/L) were statistically the same.

From the simple logistic regression (Table 4), a unit rise in FPG was significantly associated with 196 mL increase in estimated blood loss and 251 g increase in birth weight. Similarly, a unit rise in 2-h postprandial glucose was significantly associated with 290 mL increase in estimated blood loss and 562 g increase in birth weight. Perineal tear (RR [relative risk] =2.91, 95% CI: 1.08–5.57) and birth asphyxia (RR = 3.24, 95% CI: 1.01–10.45) were the only significant maternal and newborn perinatal outcomes observed from the adjusted binary regression (Table 5). However, based on specific thresholds, the IADPSG/WHO guideline for 2-h OGTT was associated with large-for-gestational-age (RR = 3.36, 95% CI: 1.14–9.85). In contrast, the ≥6.1 mmol/L threshold for fasting plasma glucose used in Ghana was associated with birth asphyxia (RR = 3.19, 95% CI: 1.79–12.86) (Table 5).

### 3.4. Postpartum Glycemic Status

At 12 weeks postpartum, we located only 20 of the 70 women diagnosed with GDM. Mean FPG had reduced from 5.70 mmol/L (SD = 0.79) during pregnancy to 4.39 mmol/L (SD = 0.83) at 12 weeks after birth. Overall, 15% (*n* = 3) had impaired fasting glucose (6.1–6.9 mmol/L) whereas 5% (*n* = 1) had diabetes (FPG ≥7.0 mmol/L).

## 4. Discussion

Depending on the test tool and diagnostic criteria, 4–24% had at least one abnormal blood glucose value. Findings reaffirmed some established risk factors for GDM, such as advanced maternal age and obesity. Meanwhile, it revealed some emerging risks such as partner’s level of education, ANC in primary facilities and intake of high glycemic index foods. Perineal trauma and birth asphyxia were key obstetric outcomes. At 12 weeks postpartum, a fifth of the GDM cases remained hyperglycemic, of which 5% was suggestive of diabetes.

Although the majority of GDM cases occur in low- and middle-income countries, the prevalence in Africa is relatively lower (9.5%) [4] as seen in Ghana (9.3%) [21], South African (9.1%) [30] and Nigeria (8.6%) [31]. Yet, isolated higher rates have been reported in Tanzania (19.5%) [32], South Africa (25.8%) [33] and Morocco (23.7%) [34]. However, the use of diverse diagnostic tests and screening algorithms coupled with differences in study populations and healthcare settings pose a challenge in comparing rates, exposures, treatment effects, pregnancy outcomes and harmonizing clinical practice [13]. Another difficulty is that many of these studies do not indicate whether the reported prevalence is derived from FPG or 2-h OGTT, which are slightly variant [2]. In Tanzania, a considerable variation (14%) was found between GDM prevalence per FPG (18.3%) and 2-h OGTT (4.3%) using the IADPSG criteria [32]. Interestingly, we found a similar variation in our study (≈14.0%). In fact, from the dietary data collected, the frequency of consumption of sugar-sweetened foods (35.7%) and beverages (21.1%) during both day and night, was relatively high. Therefore, we suspect that sub-optimum adherence to test preparations, particularly the overnight fast, could account for the high prevalence from FPG test, but this is inconclusive and needs further investigation. It also affirms the importance of nutrition education in all health promotion interventions. The lowering of GDM diagnostic thresholds has generated concerns of over-diagnosing [14,15,16], unnecessary medical interventions during pregnancy [17,18] and the associated emotional stress to the woman [35]. It is envisaged that lower-level health systems in many developing countries will be unable to manage the high number of diagnosed cases. Recommendations by the WHO for health systems to contextualize the diagnostic criteria by the IADPSG to suit the needs of individual healthcare settings [19] is worth considering.

For instance, in Ghana, a higher threshold for fasting blood glucose (above 6.0 mmol/L) is used to diagnose GDM [22]. A study in Ghana found that fasting plasma glucose ≥5.6 mmol/L yielded an optimized and clinically relevant sensitivity (80%) and specificity (74%) using the IADPSG/WHO threshold for 2-h OGTT as the gold standard [25]. Furthermore, the positive predictive value was higher (35.6%) when compared to ≥5.1 mmol/L cut-off (25.2%) [25]. Women with GDM tend to have macrosomic babies, thus requiring CS [2,9] but vaginal delivery of macrosomic babies prolongs labor, traumatizes the perineum and asphyxiates the newborn [2,9,10]. However, we found a few variations in the birth outcomes depending on the test and diagnostic criteria. In comparing the groups of GDM confirmed by different criteria, 2-h OGTT ≥8.5 mmol/L was associated with large-for-gestational-age while fasting plasma glucose ≥6.1 mmol/L was associated with birth asphyxia. The physiologic interactions between fasting glucose and 2-h OGTT during pregnancy should be further explored. A stricter diagnostic threshold could be used as is done in many centers in India where 2-h OGTT is done irrespective of the women’s fasting state and GDM diagnosed if 2-h OGTT value is ≥7.8 mmol/L [36].

We found a higher risk among primary facility users. Rural dwellers in Ghana often receive ANC in primary facilities where emergency obstetric and newborn care services are limited while medical specialists are not within reach. Meanwhile, rural dwellers experience more multiparity, poverty and illiteracy [37]. The implications are longer reproductive years, more unwanted pregnancies and poor health-seeking behaviors [38]. Improving access to basic healthcare amenities for GDM screening and management in rural facilities and sensitizing the need for optimum ANC seeking behaviors is crucial. Additionally, there is a need to ensure effective monitoring, surveillance and implementation of the GDM policy in developing countries. Rather than adopt guidelines that might pose challenges to already fragile healthcare systems, it should be adapted reference to contextual circumstances.

Intake of high glycemic index foods such as roots, tubers, plantain, rice, bread, pasta and sugar-sweetened beverages, associated with high GDM risk, is typical in Ghana, [39,40] contributing to the obesity epidemic. Socio-culturally acceptable lifestyle interventions focused on diet and weight control are crucial [8]. Firstline medical nutrition therapy could be a feasible option for many primary healthcare systems since pharmacotherapy is provided only at a higher level of healthcare. Having observed a lower risk for GDM among women whose partners have attained higher levels of formal education goes to emphasize the importance of male involvement in maternal healthcare. Males are decision-makers and financiers of many indigenous households and when involved, tend to support the women in making healthy decisions [38].

### 4.1. Strengths and Limitations of This Study

In determining participants’ body mass index, first-trimester weight was used instead of pre-pregnancy weight and was complemented by mid-upper arm circumference measurement, a reliable indicator for assessing adiposity when pregnancy is advanced. Although we validated the plausibility of dietary data obtained from the food frequency questionnaire with a 24-h recall, we did not exclude any participant based on non-plausible self-reported dietary intake. A third of the pregnant women booked for GDM testing failed to attend the appointment, but that did not affect our estimates as we accounted for 50% attrition rate in the design. However, only 29% of the GDM cases could be traced at 12 weeks postpartum. Although a very low turnout, it is not entirely surprising as in many developing countries, coverage for postnatal care tends to be relatively poor compared with other maternal and child health services. Attrition bias is a major challenge in cohort studies. Where the follow-up rate is below the acceptable thresholds (60–80%), it can threaten the validity of the results. In typical social settings similar to where our study was conducted, possible reasons that could account for the low postpartum turnout are maternal feeling of being out of danger after childbirth, being occupied with newborn care, difficulty adjusting to the new caregiver role and moving in to stay with relations who are capable of supporting with the newborn’s care. It is possible that participants who did not return for testing are significantly different from those who reported. Since not all the women scheduled for the follow-up assessments returned, the findings should be interpreted only in the context of this study and not extrapolated to the entire population. In lieu of random allocation to the different GDM test, each pregnant woman had the opportunity to do all the recommended tests. Hence, any within-individual differences are likely to be random deviations which seldom affects the true results. Although not an intervention study, we had an intention to treat yet we did not obtain data on the form of treatment administered or its effectiveness on reducing basal or prandial insulin sensitivity. Women who had abnormal glycemic results were simply referred to their obstetricians meaning that where GDM was present, it was not managed according to a unified study protocol primarily as treatment was not an objective of the study. We are therefore oblivious of the kind of therapeutic support each diagnosed woman received, the glycemic control achieved and the consequent effect of management on pregnancy outcome and postpartum glycemia.

### 4.2. Implications for Clinical and Public Health Practice

Considering that the disease burden varied widely (a 20% range) according to the type of test and diagnostic criteria used and health systems vary in the package of services they are capable of providing, the choice to use one screening procedure or diagnostic criteria over another should be established in relation to a country’s health infrastructure, health policies and services. As health systems in many low- and middle-income settings are traditionally designed to treat infections, strategies for non-communicable diseases, including GDM, are limited. We support universal testing, but depending on the health setting, lower thresholds could be used for GDM classification. Strengthening GDM detection at primary healthcare levels, where basic amenities for screening are often lacking, is vital. The use of stricter diagnostic cut-offs might favor low-income contexts where health financing and access to essential drugs and other health interventions are challenging. Nonetheless, pregnancy complicated by diabetes should be considered as an opportunity to improve metabolic and cardiovascular risk besides changing unhealthy lifestyles. Health promotion interventions that tackle modifiable risk factors such as poor dietary habits and obesity are paramount. A coordinated transition of care after regular postpartum care ceases, and the integration of post-delivery glycemic monitoring into routine health care services will facilitate the detection of persistently hyperglycemic cases after delivery.

## Figures and Tables

**Table 1:** Prevalence of GDM according to some commonly used diagnostic criteria.

Diagnostic Criteria	Fasting Plasma Glucose (*n* = 446)		2-h OGTT (*n* = 435)		FPG and/or 2-h OGTT (*n* = 446)
	Cut-Off mmol/L	%	Cut-Off mmol/L	%	%
IADPSG ^a^/WHO/FIGO/ADA	5.1	23.8	8.5	9.0	26.5
1999 WHO	7.0	2.7	7.8	14.3	14.9
NICE ^b^	5.6	10.8	7.8 ^b^	14.3	20.3
CDA ^c^	5.3	16.9	9.0	5.1	18.9
ACOG/Carpenter and Coustan ^d^	5.3	16.9	8.6	7.8	20.0
ACOG/NDDG ^d^	5.8	8.3	9.2	4.4	10.6
Ghana protocol	6.1	5.8	8.5	9.0	11.9

FPG, fasting plasma glucose; IADPSG, International Association of Diabetes in Pregnancy Study Groups; WHO, World Health Organization; FIGO, International Federation of Gynecology and Obstetrics; ADA, American Diabetes Association; NICE, National Institute for Health and Care Excellence; CDA, Canadian Diabetes Association; ACOG, American Congress of Obstetricians and Gynecologists; NDDG, National Diabetes Data Group. ^a^ IADPSG criteria have been officially adopted by WHO, FIGO, ADA, Australian Diabetes in Pregnancy Society and Brazilian Society of Diabetes. GDM is diagnosed when one or both glucose values are abnormal. ^b^ The NICE cut-off for 2-h OGTT is used by the Diabetes in Pregnancy Study group in India, but they perform 2-h OGTT irrespective of the woman’s fasting state. ^c^ CDA criteria is a two-step process starting with the 50 g glucose challenge test. ^d^ ACOG recommends 2-step screening. Diagnosis requires two or more elevated values on the 3-h OGTT.

**Table 2:** Characteristics of the GDM positive and negative reference groups.

Variables	Polychotomous Sub-Groups	GDM (*n* = 70) *n* (%)	No GDM (*n* = 376) *n* (%)	*p*-Value *
Maternal age (years)	<20	4 (5.5)	31 (7.5)	0.489
	20–29	32 (43.8)	212 (51.5)	
	30–39	32 (43.9)	158 (38.0)	
	≥40	5 (6.8)	13 (3.1)	
Parity (no. of children)	None	11 (21.6)	89 (35.5)	0.173
	1 child	15 (29.4)	70 (27.9)	
	2 children	12 (23.5)	52 (20.7)	
	≥3 children	13 (25.5)	40 (16.0)	
Woman’s education	None/primary	11 (21.6)	30 (12.0)	0.196
	Secondary/vocational	33 (64.7)	182 (73.1)	
	Tertiary	7 (13.7)	37 (14.9)	
Partner’s education	None/primary *	8 (16.7)	16 (6.7)	0.032
	Secondary/vocational *	24 (50.0)	158 (65.8)	
	Tertiary	16 (33.3)	66 (27.5)	
Level of care	Health centre *	22 (30.1)	59 (14.1)	0.001
	District hospital	47 (64.4)	299 (71.7)	
	Teaching hospital	4 (5.5)	59 (14.1)	
Body mass index	Underweight	4 (7.8)	21 (8.4)	0.061
	Normal weight *	24 (47.1)	158 (63.2)	
	Overweight *	19 (37.3)	50 (20.0)	
	Obese	4 (7.8)	21 (8.4)	
Caloric intake ^a^	Low	36 (54.5)	198 (57.9)	0.022
	Moderate	12 (18.2)	81 (23.7)	
	High	18 (27.3)	63 (18.4)	

* Bonferroni adjusted *p*-values show the column proportions which differed significantly. ^a^ Caloric intake was estimated based on glycemic index of habitually consumed foods. Habitual intake of more than four foods that contributed >70% glycemic index (GI) per day was classified as high caloric intake; habitual daily intake of 3–4 high GI foods was classified as moderate caloric intake, and habitual daily intake of 1–3 high GI foods was classified as low caloric intake.

**Table 3:** Socio-demographic, health and nutritional status indicators and the risk factors for gestational diabetes mellitus.

Risk Categories	Dichotomized Exposure Variables	Cochran-Mantel-Haenszel Test			Unconditional Binary Logistic Regression			
		GDM ^a^ (*n* = 70) *n* (%)	No GDM (*n* = 376) *n* (%)	*p*-Value	Crude Model		Adjusted Model	
					uOR	95% CI	aOR	95% CI
Socio-demographic data	Age >35 years	16 (23.9)	46 (12.0)	0.019	2.29	1.21–4.36	4.06	0.58–8.73
	Unmarried	13 (28.3)	65 (27.3)	0.859	1.05	0.52–2.12	-	-
	Rural residency	14 (28.6)	71 (29.1)	0.941	0.98	0.49–1.92	-	-
	Low education: woman	11 (21.6)	30 (12.0)	0.077	2.01	0.93–4.33	-	-
	Low education: partner	8 (16.7)	16 (6.7)	0.039	2.80	1.12–6.97	-	-
	Primary-level facility	16 (31.4)	38 (15.1)	0.009	2.56	1.29–5.08	-	-
Anthropometric indicators	Overweight/obese	13 (20.0)	39 (10.7)	0.041	2.08	1.04–4.16	2.13	1.13–4.03
	Weight >90 kg ^b^	4 (6.1)	11 (3.0)	0.182	2.08	0.64–6.75	-	-
	Height <150 cm ^b^	7 (13.7)	26 (10.4)	0.466	1.37	0.56–3.35	-	-
	High weight gain ^c^	12 (24.0)	51 (20.6)	0.574	1.21	0.59–2.49	-	-
	MUAC >30 cm ^d^	22 (34.9)	80 (21.3)	0.024	1.99	1.12–3.52	2.97	1.31–5.58
Obstetric history	Parity >3 children	8 (12.9)	22 (6.2)	0.066	2.25	0.95–5.31	2.42	0.39–4.75
	Gravida >5 pregnancies	5 (7.9)	19 (5.0)	0.365	1.63	0.58–4.53	-	-
	Prior macrosomia >4 kg	1 (16.7)	8 (18.6)	0.909	2.87	0.09–8.56	-	-
	Prior neonatal death	5 (10.2)	21 (8.0)	0.576	1.32	0.47–3.67	4.06	0.88–18.87
	Prior cesarean section	10 (20.0)	43 (16.3)	0.539	1.28	0.59–2.75	1.15	0.33–4.03
	History of abortions	18 (50.0)	62 (32.0)	0.040	2.13	1.04–4.37	4.01	1.09–14.76
	Multiple pregnancies	2 (4.0)	7 (2.7)	0.439	1.51	0.31–7.49	-	-
Medical conditions	Diabetes in family	5 (7.5)	24 (6.3)	0.787	1.20	0.44–3.26	1.50	0.31–7.31
	Family hypertension	7 (13.7)	22 (8.8)	0.296	1.65	0.67–4.11	1.21	0.34–4.36
	Glycosuria ^e^	4 (5.5)	11 (2.6)	0.171	2.14	0.66–6.91	3.65	0.76–17.42
	Hypertension	9 (17.6)	47 (18.7)	0.989	1.93	0.42–2.04	-	-
	Preeclampsia	6 (9.1)	6 (1.6)	0.004	6.23	1.15–19.96	3.98	0.50–31.42
	Antepartum depression	13 (32.5)	60 (26.2)	0.442	1.36	0.66–2.80	-	-
	Dyslipidemia ^f^	8 (15.7)	63 (25.3)	0.153	0.55	0.25–1.23	0.91	0.16–5.11
	Malaria infection	5 (12.5)	14 (6.0)	0.170	2.25	0.76–6.62	-	-
	HIV positive	2 (5.1)	2 (0.9)	0.082	5.84	0.79–42.74	-	-
Nutritional status	Anaemia (Hb < 11 g/dL)	24 (60.0)	130 (55.6)	0.365	1.20	0.61–2.37	-	-
	High caloric intake ^g^	18 (28.6)	56 (18.5)	0.080	1.76	1.95–3.28	2.91	1.05–8.07

GDM, Gestational Diabetes Mellitus; uOR and aOR, unadjusted and adjusted odds ratios. Model summary: observations = 358; Prob > Chi^2^ = 0.0116; Log likelihood = −87.904; Pseudo R^2^ = 0.2438. ^a^ GDM defined as 2-h OGTT ≥8.5 mmol/L and/or the fasting plasma glucose ≥5.6 mmol/L. ^b^ Weight and height were measured in the first trimester. ^c^ Maternal weight was measured monthly. Change in weight was high if above the threshold for the BMI category. ^d^ MUAC (mid-upper arm circumference) was measured once per trimester. ^e^ Glycosuria includes trace and 1+ to 5+ dipstick glucose at any one-time point during pregnancy. ^f^ Dyslipidemia refers to total cholesterol >7.73 mmol/L, high-density lipoprotein cholesterol <1.34 mmol/L, low-density lipoprotein cholesterol >4.76 mmol/L and triglycerides >4.31 mmol/L. ^g^ High caloric intake defined as habitual intake of high glycemic index foods ≥5 per day. Main high GI foods consumed included white bread, polished rice, processed cassava and corn meals, ripe plantain, table sugar, pasta, pineapple, watermelons and soda drinks.

**Table 4:** Simple linear regression showing the coefficients of a unit rise in fasting plasma glucose and 2-h OGTT concentration on maternal and perinatal outcomes.

Maternal and Newborn Outcomes	Fasting Plasma Glucose Values			2-h OGTT Values		
	Coef._crude_	95% CI	*p*-Value	Coef._crude_	95% CI	*p*-Value
Cesarean section *	0.185	−0.087, 0.457	0.183	0.330	−0.140, 0.801	0.168
Episiotomy *	−0.235	−0.601, 0.130	0.207	−0.490	−1.121, 0.140	0.127
Perineal tear *	0.204	−0.168, 0.575	0.281	0.143	−0.506, 0.793	0.664
Preeclampsia *	0.087	−0.193, 0.368	0.541	0.149	−0.339, 0.637	0.548
Prolong labour	0.028	−0.098, 0.155	0.660	0.077	−0.026, 0.122	0.200
Est. blood loss	0.196	0.087, −0.306	0.001	0.290	0.010–0.482	0.003
Hemoglobin	0.024	−0.065, 0.114	0.592	0.043	−0.105, 0.193	0.563
Gestational age	0.056	−0.004, 0.116	0.067	0.034	−0.072, 0.140	0.529
Birth weight	0.251	0.008, 0.494	0.043	0.562	0.141, 0.983	0.009
Birth length	0.001	−0.034, 0.036	0.969	0.003	−0.059, 0.065	0.923
Head circumference	0.056	−0.001, 0.114	0.056	0.043	−0.059, 0.147	0.405
Apgar at 5 min	−0.036	−0.119, 0.064	0.558	−0.064	−0.236, 0.072	0.296
Ponderal index ^a^	0.159	−0.030, 0.349	0.100	0.273	−0.060, 0.607	0.108
Newborn glucose	0.058	−0.156, 0.273	0.583	0.029	−0.420, 0.478	0.897
Resuscitation *	0.172	−0.081, 0.426	0.181	0.272	−0.142, 0.687	0.197
Intensive care *	−0.286	−0.881, 0.307	0.343	−0.734	−1.757, 0.288	0.158
Birth asphyxia *	0.850	−0.461, 2.163	0.203	0.457	−1.792, 2.706	0.690
Perinatal death ^b,^*	0.719	−0.353, 1.792	0.188	0.645	−1.193, 2.484	0.490

* These are categorical variables and the rest are continuous variables. The birth outcomes reported above were diagnosed or classified using case definitions by the World Health Organization which have been adopted as standard clinical practice in Ghana. Preeclampsia is defined as concomitant hypertension and proteinuria with/without edema. ^a^ Ponderal Index computed as fetal weight (g)/length (cm^3^). ^b^ Perinatal death includes both macerated and fresh cases.

**Table 5:** Relative risk for adverse pregnancy outcomes associated with GDM using different diagnostic criteria.

Maternal and Newborn Outcomes	FPG ≥ 5.1 mmol/L ^a^		2-h OGTT ≥ 8.5 mmol/L ^a^		FPG ≥ 6.1 mmol/L ^b^		FPG ≥ 5.6 mmol/L and/or 2-h OGTT ≥ 8.5 mmol/L ^c^		FPG ≥ 5.6 mmol/L and/or 2-h OGTT ≥ 8.5 mmol/L ^d^	
	uRR	95% CI (*p*-Value)	uRR	95% CI (*p*-Value)	uRR	95% CI (*p*-Value)	uRR	95% CI (*p*-Value)	aRR	95% CI (*p*-Value)
Cesarean section	1.84	0.98–3.46 (0.057)	1.44	0.57–3.62 (0.434)	1.15	0.36–3.69 (0.806)	1.70	0.84–3.44 (0.138)	1.88	0.96–3.67 (0.063)
Perineal tear	1.82	0.77–4.31 (0.171)	1.30	0.348–4.87 (0.694)	2.11	0.53–8.35 (0.287)	1.90	0.72–4.97 (0.189)	2.90	1.08–5.56 (0.043)
PPH ^e^	1.26	0.23- 6.68 (0.786)	3.72	0.69–19.80 (0.123)	2.47	0.28–21.78 (0.414)	1.82	0.35–9.41 (0.473)	4.65	0.31–9.58 (0.265)
Preterm	0.90	0.28–2.87 (0.860)	0.44	0.05–3.54 (0.448)	1.07	0.12–8.87 (0.950)	0.92	0.25–3.38 (0.912)	0.73	0.20–2.61 (0.856)
LGA ^f^	1.72	0.71–4.19 (0.226)	3.36	1.14–9.85 (0.027)	3.56	0.89–14.28 (0.072)	1.63	0.60–4.38 (0.331)	2.66	0.86–5.04 (0.254)
Resuscitated	1.07	0.57–2.01 (0.821)	0.66	0.23–1.86 (0.437)	0.88	0.27–2.88 (0.842)	1.28	0.63–2.62 (0.489)	2.90	0.93–9.01 (0.065)
Birth asphyxia ^g^	1.67	0.21–2.06 (0.490)	1.96	1.21–4.39 (0.963)	3.19	1.79–12.86 (0.042)	1.61	0.32–8.13 (0.495)	3.24	1.01–10.44 (0.039)
Macrosomia (≥4 kg)	1.50	0.36–6.20 (0.569)	2.80	0.55–14.29 (0.213)	2.05	0.24–17.55 (0.509)	1.37	0.27–6.85 (0.695)	-	-
NICU	0.31	0.03–2.53 (0.278)	0.90	0.36–2.24 (0.822)	1.80	0.21–15.15 (0.589)	0.51	0.06–4.14 (0.530)	-	-
Perinatal death	1.48	0.13–16.63 (0.748)	2.38	0.21–26.82 (0.482)	7.96	0.68–92.62 (0.097)	2.38	0.21–26.82 (0.482)	-	-

FPG, fasting plasma glucose; 2-h OGTT, two-hour oral glucose tolerance test; PPH, postpartum hemorrhage; LGA, large-for-gestational age; NICU, neonatal intensive care unit. ^a^ World Health Organization [19] recommendation for fasting plasma glucose and 2-h OGTT. ^b^ In Ghana, FPG cut-off is ≥6.1 mmol/L but 2-h OGTT is same as for the WHO criteria. ^c^ Criteria we propose to use in Ghana showing the ^c^ unadjusted and ^d^ adjusted regression models. ^d^ Model summary: N = 385; Prob > Chi^2^ = 0.035; Log likelihood = −51.317; Pseudo R^2^ = 0.1686. ^e^ Postpartum hemorrhage was defined as blood loss >500 mL. ^f^ Large for gestational age was computed as birth weight >90th percentile for gestational age. ^g^ Birth asphyxia diagnosed as Apgar score five minutes after birth after below four.

## Data Availability

The data presented in this study are available on request from the corresponding author. The data are not publicly available due to ethical restrictions.

## References

[1] Duncan J.M. (1882). On puerperal diabetes. Trans. Obstet. Soc. Lond..

[2] Metzger B.E., Lowe L.P., Dyer A.R. (2008). Hyperglycemia and adverse pregnancy outcomes. N. Engl. J. Med..

[3] IADPSG Consensus Panel (2010). International association of diabetes and pregnancy study groups recommendations on the diagnosis and classification of hyperglycemia in pregnancy. Diabetes Care.

[4] Cho N., Shaw J., Karuranga S. (2018). IDF Diabetes Atlas: Global estimates of diabetes prevalence for 2017 and projections for 2045. Diabetes Res. Clin. Pract..

[5] American Diabetes Association (2015). Classification and diagnosis of diabetes. Diabetes Care.

[6] Mwanri A.W., Kinabo J., Ramaiya K. (2015). Gestational diabetes mellitus in sub-Saharan Africa: Systematic review and metaregression on prevalence and risk factors. Trop. Med. Int. Health.

[7] Muche A.A., Olayemi O.O., Gete Y.K. (2019). Prevalence and determinants of gestational diabetes mellitus in Africa based on the updated international diagnostic criteria: A systematic review and meta-analysis. Arch. Public Health.

[8] Hod M., Kapur A., Sacks D.A. (2015). The International Federation of Gynecology and Obstetrics (FIGO) Initiative on gestational diabetes mellitus: A pragmatic guide for diagnosis, management, and care. Int. J. Gynecol. Obstet..

[9] Wendland E.M., Torloni M.R., Falavigna M. (2012). Gestational diabetes and pregnancy outcomes-a systematic review of the World Health Organization (WHO) and the International Association of Diabetes in Pregnancy Study Groups (IADPSG) diagnostic criteria. BMC Pregnancy Childbirth.

[10] O’Sullivan E., Avalos G., O’Reilly M. (2011). Atlantic Diabetes in Pregnancy (DIP): The prevalence and outcomes of gestational diabetes mellitus using new diagnostic criteria. Diabetologia.

[11] Eades C.E., Styles M., Leese G.P. (2015). Progression from gestational diabetes to type 2 diabetes in one region of Scotland: An observational follow-up study. BMC Pregnancy Childbirth.

[12] Kampmann U., Madsen L.R., Skajaa G.O. (2015). Gestational diabetes: A clinical update. World J. Diabetes.

[13] Agarwal M. (2018). Consensus in gestational diabetes mellitus: Looking for the holy grail. J. Clin. Med..

[14] Cundy T., Ackermann E., Ryan E.A. (2014). Gestational diabetes: New criteria may triple the prevalence but effect on outcomes is unclear. BMJ.

[15] Bolognesi M. (2015). Overdiagnosis of Gestational Diabetes Mellitus in Pregnant Woman: A Case Report. J. Women’s Health Care.

[16] Twohig H., Hodges V., Mitchell C. (2018). Pre-diabetes: Opportunity or overdiagnosis?. Br. J. Gen. Pract..

[17] Glasziou P. (2017). Sustainable health care and the problem of overdiagnosis. Pathology.

[18] Moynihan R. (2016). Preventing overdiagnosis–winding back the harms of too much medicine. Port. J. Nephrol. Hypert..

[19] World Health Organization (2013). Diagnostic Criteria and Classification of Hyperglycaemia First Detected in Pregnancy.

[20] Silver R.M. (2019). GDM: More diabetes, more good or more harm?. BJOG Int. J. Obstet. Gynaecol..

[21] Oppong S.A., Ntumy M.Y., Amoakoh-Coleman M. (2015). Gestational diabetes mellitus among women attending prenatal care at Korle-Bu Teaching Hospital, Accra, Ghana. Int. J. Gynecol. Obstet..

[22] Ministry of Health Ghana (2017). Standard Treatment Guidelines. Accra Ghana Ministry of Health and Ghana National Drugs Programme (GNDP).

[23] Beyuo T., Obed S.A., Adjepong-Yamoah K.K. (2015). Metformin versus Insulin in the Management of Pre-Gestational Diabetes Mellitus in Pregnancy and Gestational Diabetes Mellitus at the Korle Bu Teaching Hospital: A Randomized Clinical Trial. PLoS ONE.

[24] Ghana Health Service (2017). Family Health Division Annual Report 2016.

[25] Agbozo F., Amardi-Mfoafo J., Dwase H. (2018). Nutrition knowledge, dietary patterns and anthropometric indices of older persons in four peri-urban communities in Ga West municipality, Ghana. Afr. Health Sci..

[26] National Institute for Health and Care Excellence (2015). Diabetes in Pregnancy. Management of Diabetes and Its Complications from Preconception to the Postnatal Period. NICE Guideline 3 Methods, Evidence and Recommendations.

[27] Diabetes Canada Clinical Practice Guidelines Expert Committee (2018). Diabetes Canada 2018 clinical practice guidelines for the prevention and management of diabetes in Canada. Can. J. Diabetes.

[28] Committee on Practice Bulletins—Obstetrics (2018). ACOG practice bulletin No. 190, gestational diabetes mellitus. Obstet. Gynecol..

[29] World Health Organization (2006). Definition and Diagnosis of Diabetes Mellitus and Intermediate Hyperglycaemia: Report of a WHO/IDF Consultation.

[30] Macaulay S., Ngobeni M., Dunger D.B. (2018). The prevalence of gestational diabetes mellitus amongst black South African women is a public health concern. Diabetes Res. Clin. Pract..

[31] Olagbuji B.N., Atiba A.S., Olofinbiyi B.A. (2015). Prevalence of and risk factors for gestational diabetes using 1999, 2013 WHO and IADPSG criteria upon implementation of a universal one-step screening and diagnostic strategy in a sub-Saharan African population. Eur. J. Obstet. Gynecol. Reprod. Biol..

[32] Njete H., John B., Mlay P. (2018). Prevalence, predictors and challenges of gestational diabetes mellitus screening among pregnant women in northern Tanzania. Trop. Med. Int. Health.

[33] Adam S., Rheeder P. (2017). Screening for gestational diabetes mellitus in a South African population: Prevalence, comparison of diagnostic criteria and the role of risk factors. SAMJ South Afr. Med. J..

[34] Utz B., Assarag B., Smekens T. (2018). Detection and initial management of gestational diabetes through primary health care services in Morocco: An effectiveness-implementation trial. PLoS ONE.

[35] Feighan C., Devine H., Daniel U. (2017). The emotional journey of gestational diabetes. Lancet Diabetes Endocrinol..

[36] Government of India (2018). Diagnosis and Management of Gestational Diabetes Mellitus. Technical and Operational Guidelines: Ministry of Health and Family Welfare.

[37] Ghana Statistical Service (2013). 2010 Population and Housing Census, Regional Analytical Report, Volta Region.

[38] Mathes T., Jaschinski T., Pieper D. (2014). Adherence influencing factors—A systematic review of systematic reviews. Arch. Public Health.

[39] Galbete C., Nicolaou M., Meeks K.A. (2017). Food consumption, nutrient intake, and dietary patterns in Ghanaian migrants in Europe and their compatriots in Ghana. Food Nutr. Res..

[40] Agbozo F., Atitto P., Jahn A. (2018). Nutrient composition and dietary diversity of on-site lunch meals, and anthropometry of beneficiary children in private and public primary schools in Ghana. Nutr. Health.

